# Effect of Microgroove Structure in PDMS-Based Silicone Implants on Biocompatibility

**DOI:** 10.3389/fbioe.2021.793778

**Published:** 2022-01-20

**Authors:** Yao Chen, Xin Zhou, Shuqing Huang, Yujie Lan, Rongshuai Yan, Xiaohua Shi, Xiang Li, Yiming Zhang, Zeyuan Lei, Dongli Fan

**Affiliations:** Department of Plastic and Cosmetic Surgery, Xinqiao Hospital, The Army Medical University, Chong Qing, China

**Keywords:** microgrooves, silicone implants, fibrosis, capsular formation, foreign body reaction

## Abstract

Capsule and capsule contracture around implants are important concerns in a clinic. The physical topology of the material surface regulates the formation of the capsule, but the specific regulatory mechanism is unclear. In this study, four types of silicone implant materials with different microgroove structures (groove depths of 10 and 50 μm and widths of 50 and 200 μm) were constructed using lithography to form different gradient surface topologies. Mass spectrometry, Cell Counting Kit-8, 5-ethynyl-2′-deoxycytidine (EdU), enzyme-linked immunosorbent assay, western blot, immunofluorescence, and immunohistochemistry were used to explore the changes in protein adsorption, cell adhesion, cell proliferation, and collagen deposition on the surface of the materials. At the same time, RNA-seq was used to detect transcriptome differences caused by different structures. Furthermore, collagen deposition and capsule formation were observed in the rats. The groove structure was observed to significantly increase the surface roughness of the material. The deeper groove and the narrower width of the polydimethylsiloxane would increase the surface roughness of the material and the surface water contact angle but reduce the total amount of adsorbed protein in the first two hours. *In vitro* cell experiments revealed that microtopology affected cell proliferation and adhesion and regulated collagen secretion. Further analysis indicated the deeper and narrower groove (group 50–50) on the surface of the material caused more evident collagen deposition around the material, forming a thicker envelope. Surface roughness of the material was thus related to collagen deposition and envelope thickness. The thickness of the envelope tissue around smooth materials does not exceed that of the materials with surface roughness. In conclusion, the narrower and deeper grooves in the micron range exhibited poor histocompatibility and led to formation of thicker envelopes around the materials. The appropriate grooves can reduce envelope thickness.

## Introduction

Following the development of medicine, implantable medical devices and tissue or organ transplant substitutes have revolutionized modern medicine. Implants have a wide range of clinical applications, including vital sign detection (Nichols et al., 2013), tissue repair and reconstruction (Kaoutzanis et al., 2019; Prasad et al., 2019; Sharma et al., 2019), cardiac pacemakers (Rosen et al., 2011), and drug delivery systems (Saghazadeh et al., 2018; Liang et al., 2021). Such implants are undoubtedly important for the patients' health but are also foreign devices in the body and thus trigger a tissue reaction, termed the foreign body reaction, which includes protein adsorption on the implant surface, infiltration of inflammatory cells, fusion of macrophages and foreign body giant cells, activation of fibroblasts, and the formation of the fiber capsules (Anderson et al., 2008; Klopfleisch and Jung, 2017). Fibrosis of the tissue around the implant can lead to adhesion of the surrounding tissues, such as adhesion of large blood vessels (Keiler et al., 2017). This can alter local anatomy and affect the function of the implant. In severe cases, such as fibrosis of breast prosthesis implants, capsular contracture can cause deformation of the organs and necessitate an additional surgical procedure for the patient (Puskas and Luebbers, 2012; Calobrace et al., 2018; Bengtson, 2021). Current long-term methods for maintaining the biocompatibility of biomedical implants include continuous application of anti-inflammatory drugs, glucocorticoids, and surface modification of biological materials (Anderson et al., 2008; Jaggessar et al., 2017; Rasouli et al., 2018). The addition of continuous drugs, such as anti-inflammatory drugs and glucocorticoids, has additional toxic and side effects on patients, further weighing them down. In recent years, owing to the maturity of technical conditions, modifying the surface physical properties of materials (especially the change in topological structure) has become possible.

Modification of the surface physical properties has exhibited significant advantages over the use of traditional drugs. Advances in modern technology have enabled an increasing number of methods for achieving material surface modification, such as surface deposition coating and film (Dalby et al., 2006), or surface *in-situ* modification. Among them, *in-situ* surface modification includes plasma injection/deposition (Li et al., 2017), electrochemical methods (Dalby et al., 2006), mechanical methods such as laser etching (Blttler et al., 2006), and mechanical grinding (Singh et al., 2013). As industrial technologies have progressed, it has become possible to modify the surfaces of materials with micro–nano sizes. Lithography is one of the first micro-manufacturing technologies applied to the biological field and can be used to modify surfaces with specific morphologies and nanostructures while preserving their chemical properties (Yang et al., 2009). As a standard process for manufacturing integrated circuits in the semiconductor industry, lithography has a high precision and can produce high-precision graphics single chips for cell surface interactions (Kunzler et al., 2006). Although lithography is easily limited by the wavelength of light, the type of photosensitive resist used, and imaging constraints (Gardner et al., 2006), it can quickly, cost-effectively, and continuously manufacture structures of any desired shape. The combination of lithography and related micro–nano processing technology can be used in research. However, it is unclear how the surface topological properties affect cells and whether there are differences in the effects of different topological structures on cells.

In this study, we used a polydimethylsiloxane (PDMS)-based silicone implant, a common material for medical implants, to construct microgrooves of different sizes on the surface to achieve different topological structures and study their effects on cells and tissues as well as their effects on tissue fibrosis to identify suitable surface and interface properties to meet the characteristics required by different functions of the implant material in the body.

## Materials and Methods

### Material Preparation

CNC precision carving equipment was used to process 10 cm × 10 cm square grooves and PMMA (polymethyl methacrylate) molds with a groove depth of 0.5 mm. The PDMS prepolymer was cured on a PMMA mold and consolidated in a constant-temperature drying oven at 60–75°C for 1 h to obtain a smooth PDMS film, named group 0–0.

**FIGURE 1:**
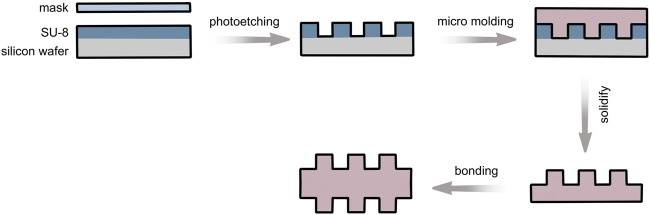
Pattern diagram of groove structure formation on the material surface.

AutoCAD 2007 (Autodesk, United States) was used to design four different sizes of microgroove structures using depth and width as indicators; the depths were 10 and 50 μm and the widths were 50 and 200 µm. The corresponding silicon wafer was selected as the mold. The homogenizing machine was used to elevate the SU-8 photoresist to the corresponding groove height, and SU-8 photoresist was prebaked on a hot plate, cooled slowly on the hot plate, and exposed by contact on the Karl Suss MA6 UV lithography machine to obtain the photoresist structure. Then, SU-8 photoresist microstructure was solidified on the hot plate at 150°C–200°C to obtain the mold. The treated bubble-free PDMS prepolymer was then solidified on the mold, dried, and peeled off after solidification to obtain four types of microgroove PDMS substrate layers. The same two pieces of PDMS with groove structures were directly bonded by plasma oxidation and irreversible sealing (Figure 1). PDMS with a double-sided groove structure was obtained by strengthening in a constant-temperature drying box at 60–75°C for 1 h.

### Surface Characterization

The samples were washed in an ultrasonic bath, dried at room temperature, and cut to a size of 1 cm × 1 cm for characterization tests. The water contact angles of all the samples were measured by a drop shape analysis system (OCA20, Dataphysics, Germany). About 10 ul of distilled water was used as the medium and dropped onto the surface of each material. Contact angles were measured in the sessile mode at 25°C, with five measurements taken for each sample. Samples were then sputtered with gold, and scanning electron microscopy (Sigma500, ZEISS, Germany) was used with an accelerating voltage of 5 keV to observe the surface morphology of each material. Furthermore, laser scanning confocal microscopy (OLS4100, OLYMPUS, Japan) was employed to observe surface micromorphology and measure surface roughness, with each sample observed in triplicate.

### Preparation of Extracellular Fluid

After injecting 5 ml of 3% pentobarbital sodium into the ear of the rabbit, the limbs were fixed after the rabbits were anesthetized, the sternum was cut through the median chest incision, and the heart was exposed. The supernatant was poured into the centrifuge at 3,000×*g* for 15 min and then stored at −20°C for later use.

### Cell Culture

In this study, human foreskin fibroblast 1 (HFF-1) cells were cultured in a complete medium with Dulbecco's Modified Eagle Medium, high-sugar, with 15% fetal bovine serum (FBS) and 1% penicillin in a 37°C, 5% CO_2_ incubator. Each group of materials was cut to a 96-well size and spread on the bottom of the culture plate for subsequent experiments. The HFF-1 cells were seeded in a 96-well plate with materials at a density of 2 × 10^4^ cells/well for the Cell Counting Kit-8 (CCK8) and 5-ethynyl-2′-deoxycytidine (EdU) detection of cell proliferation and seeded at 10^5^ cells/well in a 60-mm cell culture dish with materials for extracting protein and RNA for subsequent experiments.

### Protein Adsorption Experiment

Each group of materials with a double-sided groove structure was cut to a size of 1 cm × 1 cm and placed at the bottom of the centrifuge tube. Then 1 ml of the corresponding 10% FBS and 1% bovine serum albumin (BSA) diluents were added to each group in turns. After adsorption for 30 min, 1 h, 6 h, 12 h, and 24 h at 37°C, discarding protein solutions, adding 400 μL TBST, and shaking the elution slowly for 1.5 h on a shaker, the eluted protein concentration was tested with the BCA kit.

The materials of each group were soaked in extracellular fluid, adsorbed at 37°C for 2 h, rinsed gently with PBS, and eluted using 400-µL TBST shaker for 1.5 h. The supernatant protein solution was separated and tested using a BCA kit. The protein solution was then added to 50 ul of 50 mM ammonium bicarbonate and 1 ul of 1 M dithiothreitol and incubated at 56°C for 30 min, after which it was alkylated by adding 5 ul of 0.5 M iodoacetamide in the dark at 37°C for 10 min. The sample was then precipitated using prechilled acetone at a volumetric ratio of 6:1 of acetone to sample at −20°C overnight. After cryogenic centrifugation, the precipitate was washed twice with cold acetone and then resuspended in 50 mM ammonium bicarbonate. Finally, the proteins were digested with trypsin at a substrate/enzyme ratio of 50:1 (w/w) at 37°C for 18–20 h.

After the peptides were separated by reverse-phase chromatography (U3000nano, Thermo Fisher Scientific, United States), the samples were analyzed by LC-MS/MS analysis on a tandem mass spectrometer (LTQ Orbitrap Velos Pro, Thermo Fisher Scientific, United States). Tandem mass spectra were searched against the SwissProt Rabbit database concatenated with a reverse decoy database. The differentially expressed proteins were screened, and the genes related to the differentially expressed proteins were analyzed by the Gene Ontology (GO) enrichment analysis.

### Cell Viability Test

Each group of materials was spread on the bottom of a 96-well plate, and 2 × 10^4^ cells/well of HFF suspension was added to each well, then incubated in an incubator for 24, 48, and 72 h, and the CCK8 reagent and medium were mixed at a ratio of 1:10. After discarding the culture medium, 200 µL of the working solution was added to each well. After incubating at 37°C for 30 min, 100 µL from each well was obtained to measure the optical density value using a plate reader (Varioskan Flash, Thermo Fisher Scientific) at 520 nm, and the cell proliferation activity of each group was compared.

EdU can be used to detect newly synthesized cellular DNA and can also be used in combination with nuclear markers such as Hoechst for double labeling and detection of the cell proliferation rate. For detection, the cells were first plated in wells of a 96-well plate with the materials and cultured for 24 h, stained according to the Cell Light EdU Apollo 488 *In Vitro* Kit (Ribobio, China, C10310-3), and then diluted with a complete cell culture medium at a ratio of 5,000:1 to prepare an appropriate amount of 10 μM EdU medium. Afterward, 100 µL of the 10 μM EdU medium was added to each well and incubated for 24 h. After rinsing with PBS and fixing with paraformaldehyde for 30 min, 50 μL of 2 mg/ml glycine was added to each well, incubated for 5 min, then incubated with 0.5% Triton X-100 penetrant for 10 min, rinsed with PBS, and stained. After incubation for 30 min, the cells were rinsed with PBS again and stained with DAPI for 10 min. Fluorescence microscopy was used to observe and capture images. ImageJ software was used for counting.

### Immunofluorescence

Each group of materials was spread on the bottom of a 24-well plate, and 2 ml of 4 × 10^4^ cells/ml HFF suspension was added to each well and incubated for 48 h in an incubator. The samples were first fixed with paraformaldehyde for 30 min, and then incubated with 0.5% Triton X-100 for 10 min for permeabilization. Samples were then incubated with the primary antibody vinculin (26520-1-AP; Proteintech, China) diluted at a ratio of 1:500 overnight. Subsequently, Alexa Fluor 647–labeled goat anti–rabbit antibody (A-21244, Invitrogen, United States) diluted at a ratio of 1:500 was added and incubated for 1.5 h at 25°C, followed by a phalloidin working solution Phalloidin-iFluor 488 Reagent (ab176753, abcam, United States) diluted at a ratio of 1:1,000 and incubated at 25°C for 1.5 h. The nuclei were stained with DAPI for 10 min, mounted with an anti-fluorescence quencher, and observed under a laser confocal microscope.

### Western Blot

The materials of each group were spread in a 60-mm cell culture dish, inoculated with 3 × 10^5^ cells/well, cultured for 3 and 7 days after trypsin digestion, and centrifuged. The RIPA lysis buffer was added after lysis on ice and centrifuged at 12,000 rpm and 4°C for 10 min. The supernatant was collected as the total protein solution, and the total protein concentration was measured using the BCA method. The protein solution was then stored at −20°C after denaturation in boiling water for 15 min. An 8% separation gel and a 5% concentrated gel were prepared for protein separation, and the proteins were separated by gel electrophoresis run at 120 and 75 V, respectively. A methanol-activated PVDF membrane was used with a constant flow of 300 mA in membrane transfer solution for half an hour. The PVDF membrane was then blocked on a shaker at room temperature with skim milk for 30 min. The primary antibody was incubated overnight at 4°C, the PVDF membrane was washed thrice with TBST, and the secondary antibody was diluted at a ratio of 1:3,000, incubated for 30 min at 25°C, and exposed and developed by chemiluminescence after the membrane was washed with TBST. The primary antibodies used were GAPDH (1:1,000, GB12002, Servicebio, China), matrix metalloproteinase-1 (MMP-1) (1:1,000, 10,371-2-ap, Proteintech, China), MMP-2 (1:1,000, 10,373-1-ap, Proteintech, China), α-SMA (1:1,000, 14,395-1-ap, Proteintech, China), PCNA (1:1,000, GB11010, Servicebio, China), and MMP-9 (1:1,000, GB11132, Servicebio, China), and the intensity of each blot are measured by Quantity One software and was normalized to the loading control (GAPDH).

### Enzyme-Linked Immunosorbent Assay

The supernatant of the cells cultured on the surface of each group of materials for 3 and 7 days was collected and centrifuged at 2,000 rpm for 20 min at 4°C to remove cell debris, and the supernatant was used for MMP-2 protein quantification and determination of hydroxyproline (Hyp) content. The concentration of MMP-2 in the supernatant of the culture medium was determined by enzyme-linked immunosorbent assay (ELISA; bsk11038, Bioss, China) following the manufacturer's instructions. After adding the digestive solution, the samples were incubated in a 37°C water bath for 3 h and added following the instructions on the Hyp kit (A030-1-1, Jiancheng Bioengineering Institute, Nanjing, China).

### Ribonucleic Acid-Seq Analysis

Total RNA was extracted from the cultured HFF-1 cells using the TRIzol reagent kit (Invitrogen, Carlsbad, CA, United States) according to the manufacturer’s instructions. RNA quality was analyzed using the Agilent 2100 Bioanalyzer (Agilent Technologies, Palo Alto, CA, United States) and checked using RNase-free agarose gel electrophoresis. After the total RNA had been extracted, eukaryotic mRNA was enriched using Oligo(dT) beads, while prokaryotic mRNA was enriched by removing rRNA using the Ribo-Zero™ Magnetic Kit (Epicenter, Madison, WI, United States). Then, the enriched mRNA was fragmented into short fragments using a fragmentation buffer and reverse transcribed into cDNA with random primers. Second-strand cDNA was synthesized using DNA polymerase I, RNase H, dNTPs, and a buffer. Then following the purification of cDNA fragments, as well as end repair and poly(A) addition, the samples were ligated to Illumina sequencing adapters. The ligation products were size selected by agarose gel electrophoresis, then PCR amplified, and sequenced using Illumina HiSeq 2500 by Gene Denovo Biotechnology Co. (Guangzhou, China).

### Implantation Experiment in Rats

The animal experimental protocols used in this study were approved by the Institutional Animal Care and Use Committee of the Army Medical University, China. This study was conducted in compliance with the Guide for the Care and Use of Laboratory Animals. Each material was cut into 1 cm × 1 cm pieces. After ultrasonic cleaning for 1 h, the materials were disinfected with 75% alcohol and rinsed with sterile PBS. After female SD rats were anesthetized with 0.1% pentobarbital sodium, five independent incisions approximately 1 cm in size were made subcutaneously on the backs of the rats under sterile conditions. Five groups of materials were implanted into the rats. The rats were killed after 3 and 6 months. The materials and their surrounding tissues were removed and stained with hematoxylin and eosin (HE) and Movat stains. The inflammatory reaction, envelope thickness, and collagen deposition around the materials were observed, and the expression mechanisms of collagen I, collagen III, and elastin were observed *via* immunohistochemistry.

### Statistical Analysis

Data are reported as the mean ± standard deviation. Multiple comparisons were analyzed using a one-way ANOVA test followed by the Holm–Sidak *t* test; *p* < 0.05 was considered statistically significant. For the proliferation data, each group had *n* = 5, and for the gene expression data, each group had a technical replicate of *n* ≥ 3.

## Results

### Different Surface Topological Structures Form Different Roughness and Hydrophobicity

**FIGURE 2:**
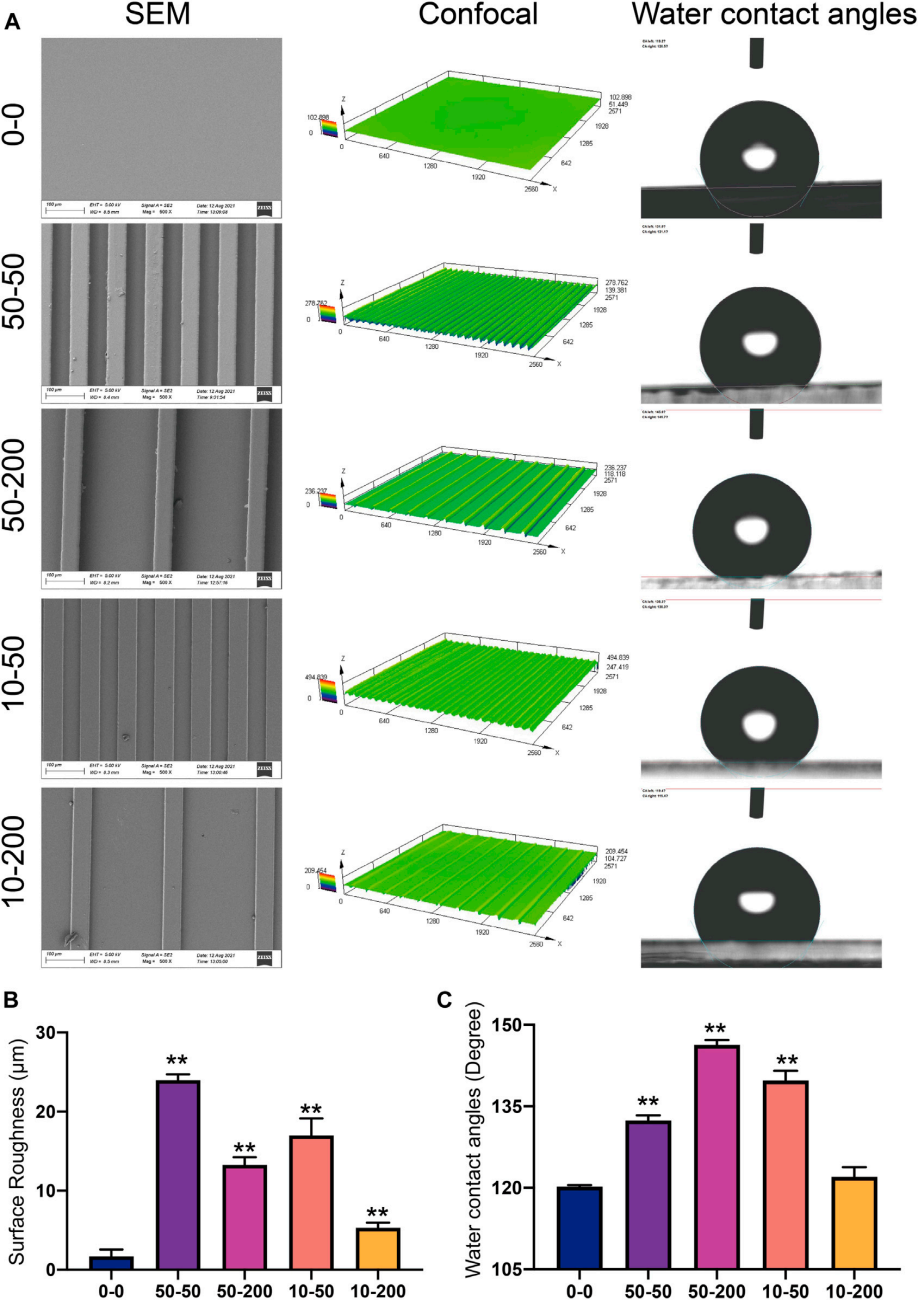
Surface characterization. **(A)** Representative images of a scanning electron microscope, laser confocal microscope, and water contact angle. **(B)** Statistical analysis of surface roughness (Sa) measured using a laser confocal microscope. **(C)** Statistical analysis of the water contact angle. Data comparisons were implemented utilizing one-way ANOVA, and the values are expressed as mean ± standard deviation (SD). *n* = 3, **p < 0.01 *vs* 0–0.

The microstructure of the surface of each group was examined using a scanning electron microscope. Except for group 0–0, apparent groove-like structures were visible on the surfaces of the other groups. The widths of the bottom of the groove in groups 50–50 and 10–50 was 50 µm. The widths of the protruding spine were both 50 μm, and the heights of the groove were 50 and 10 μm, respectively. The widths of the bottom of the groove in groups 50–200 and 10–200 were 200 µm. The widths of the protruding spine were both 50 μm, and the groove height was 50 and 10 μm, respectively (Figure 2A). A laser confocal microscope can display the 3D morphology of the material surface. Through confocal measurement of the surface roughness of each group, it was found that the groove structure can significantly increase the material surface roughness. The deeper the material, the narrower the groove width and the greater the roughness. The roughness of groups 50–50 and 10–50 were significantly higher than that of groups 50–200 and 10–200 (Figures 2A,B). Hydrophilicity and hydrophobicity were determined by the static water contact angle on the material surface. The test results showed that after the microgroove structure was constructed on the surface of the material, the water contact angle increased. Significant differences were observed among groups 50–50, 50–200, and 10–50 and group 0–0, of which the 50–200 water contact angle was the largest, and the 10–200 water contact angle also increased compared with the control group, but the difference was not statistically significant (Figures 2A–C–C).

### Surface Topology Affects Early Protein Adhesion on the Quantities and Types

The interaction between biomaterials and biological environment produces biobehavioral effects. First, the biological functions are realized through the interaction between the material interface and biomolecules and cells in the biological environment, where the adsorption of proteins on the material surface is the primary process. We used the common pattern protein solution and extracellular fluid around the implant materials to observe differences in protein adhesion for different surface topologies. Three groups: 0–0, 50–50, and 50–200 were selected, and the differences in surface microgroove adsorbed proteins were studied by protein mass spectrometry. Bioinformatics technology was used to analyze the relationships between proteins.

**FIGURE 3:**
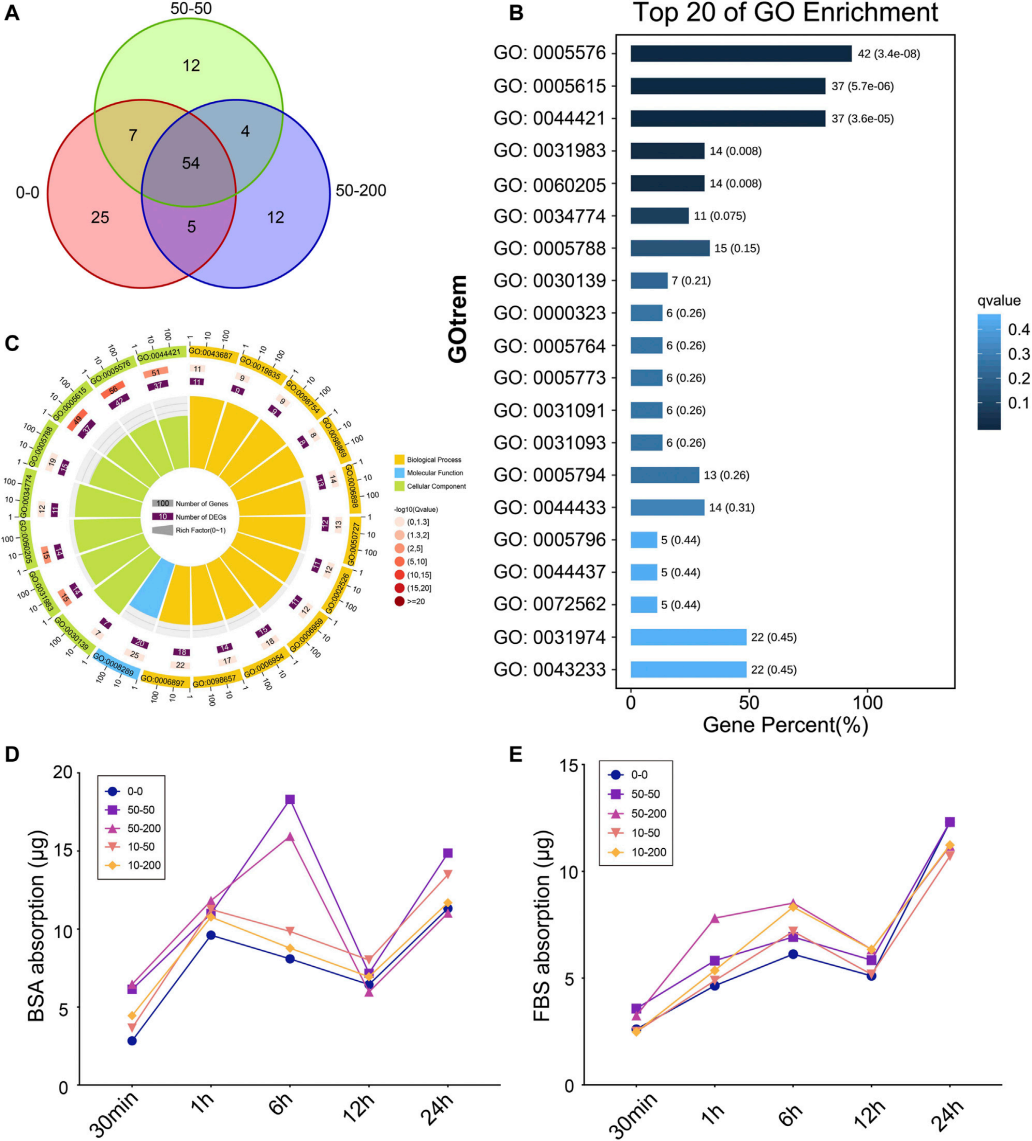
Surface topology affects protein adsorption. **(A)** Protein adsorption quantity analysis based on mass spectrometry. **(B)** Top 20 of GO enrichment analysis. **(C)** Circle maps of GO analysis. **(D)** BSA protein adsorption capacity. **(E)** FBS protein adsorption capacity.

As shown in Figure 3A, the total amount of proteins adsorbed on the surface of the three groups of materials: 0–0, 50–50, and 10–50 differed. Groups 0–0, 50–50, and 50–200 adsorbed 91, 77, and 75 proteins, respectively, 54 of which were common in the three groups. Therefore, we found that the types of adsorbed proteins of smooth materials increased significantly compared with those of grooved materials, which was contrary to the traditional concept that the increase in the adsorbed proteins is owing to an increase in the surface area of the patterned materials. A GO enrichment analysis was performed on 54 adsorbed proteins, 42 proteins of which were located in the extracellular region of the cell, which was the outer space of the outer layer of the cell, mainly referring to the products secreted or released by the cell to the blood or tissue fluid. Furthermore, 37 proteins were extracellular space proteins that contributed to the formation of the cell space (Figure 3B). Figure 3C shows that more than 50% of the same proteins adsorbed on the surface of the three groups of materials contributed to biological processes, and 40% of the proteins contributed to cellular components.

Furthermore, we selected FBS and BSA proteins to explore the changes in protein adsorption on the material surface over time. As shown in Figure 3D, in the first 6 h of BSA protein adsorption, the difference between groups was not apparent, and the curve was concentrated, except that the adsorption capacity of groups 50–50 and 50–200 increased at 6 h, and then the curve tended to be concentrated at 12 and 24 h; the quality of adsorbed protein varied slightly among the groups. As shown in Figure 3E, there was no significant difference among the groups within the first 30 min of FBS adsorption. At 1 h of adsorption, the adsorption capacity of 50–200 increased significantly, the difference in adsorption capacity decreased at 6 h, and then the protein adsorption capacity was gradually consistent at 12 and 24 h. The results showed that the difference in the protein adsorption capacity of each group was mainly reflected in the initial 6 h. Over time, the quality of the proteins adsorbed by each group gradually tended to become the same. The difference in early adsorption affected the response of cells to materials and changed the biological behavior of the cells.

### Effects of Surface Topology on Fibroblast Adhesion, Proliferation, and Collagen Formation

**FIGURE 4:**
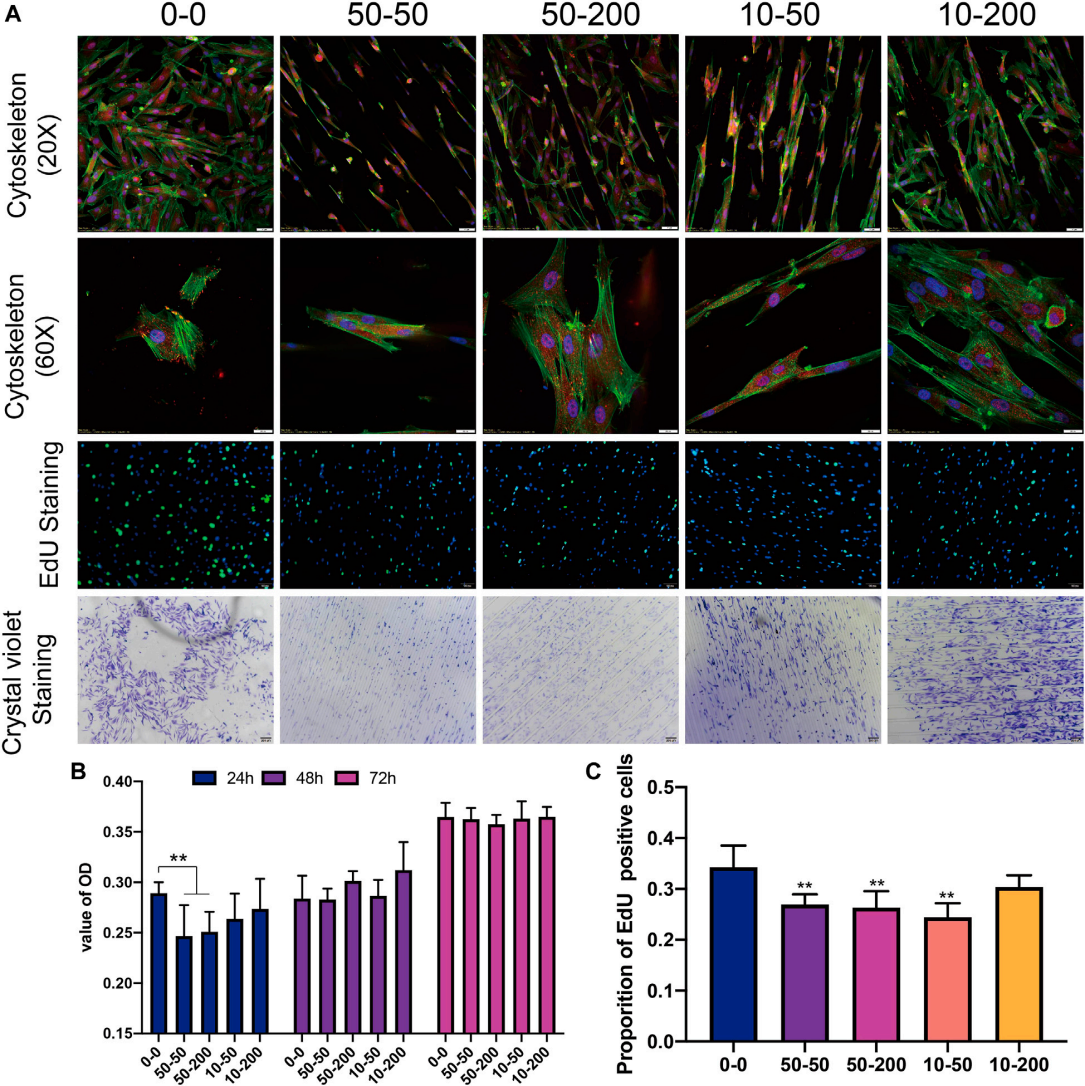
Effects of surface topology on fibroblast adhesion, proliferation. **(A)** Representative results of overall (20×) and local (60×) characteristics of cytoskeleton staining, EdU staining (scale bar is 100 μm), and cell crystal violet staining (scale bar is 200 μm). **(B)** Statistical analysis of CCK8. **(C)** EdU positive rate. Data comparisons were implemented utilizing one-way ANOVA, and the values were expressed as mean ± standard deviation (SD). *n* = 5, **p < 0.01 *vs* 0–0.

The cytoskeleton was stained with phalloidin to allow for the observation of the spreading of cells on the surface of each group of materials; the difference in the adhesion key protein vinculin was also observed. Figure 4A shows that on the group 0–0 surface, the cells spread more extensively; the spreading area was larger, the stress fibers were thicker and noticeable, and the focal adhesion expressed by vinculins was clear. On the surface of grooved materials, cells have three growth modes: extension in the groove, growth on the protruding ridge, and growth in the included angle between the groove and ridge. The cells at the bottom of the groove extended more cellular pseudopodia at both ends and were anchored on the sides of the ridges on both sides. In group 50–50, the supraspinal cells grew less, and in group 10–50 with shallow depth, the supraspinal cells grew further; cells in both groups were long spindle shaped; the cell length width ratio was large; the cell spreading area was small; the stress fibers were not noticeable; and vinculin expression was lower. However, in groups 50–200 and 10–200, the cells spread over a relatively large area, the cell length–width ratio was reduced, and the stress fibers became longer; however, the stress fiber was not as thick as in the 0–0 group. The grooves formed on the material surface hindered the formation of stress fibers to a certain extent, and the narrower the grooves, the more apparent the effect. Furthermore, the adhesion of the cells on the material surface was observed by crystal violet staining. The results showed that on the surface of group 0–0 materials, pieces of cells easily fell off during cleaning. However, on the surface of the materials with grooves, the presence of grooves prevented pieces of the cells from falling. The morphology of cells in group 50–50 was irregular, while most cells in group 10–50 exhibited a normal long spindle shape, and the morphology of cells in groups 50–200 and 10–200 was normal (Figure 4).

We detected cell DNA replication activity using EdU staining. The results of 24 h EdU activity staining showed that the proliferation of surface cells in groups 50–50 and 50–200 was significantly lower than that in the smooth group 0–0. This shows that the materials with grooves on the surface mainly affect the proliferation activity of cells in the early stage, and there is little difference between the groups following the extension of time (Figures 4A–C). After the fibroblasts were inoculated on the materials of each group and cultured, CCK8 was used to detect cell activity. After 24 h, the cell activity of groups 50–50 and 50–200 exhibited a decrease that was statistically significant. Although the activity of the other two groups decreased compared with group 0–0, the difference was not statistically significant. There was no significant difference between the experimental and control groups over the subsequent 48 and 72 h (Figures 4A,B).

**FIGURE 5:**
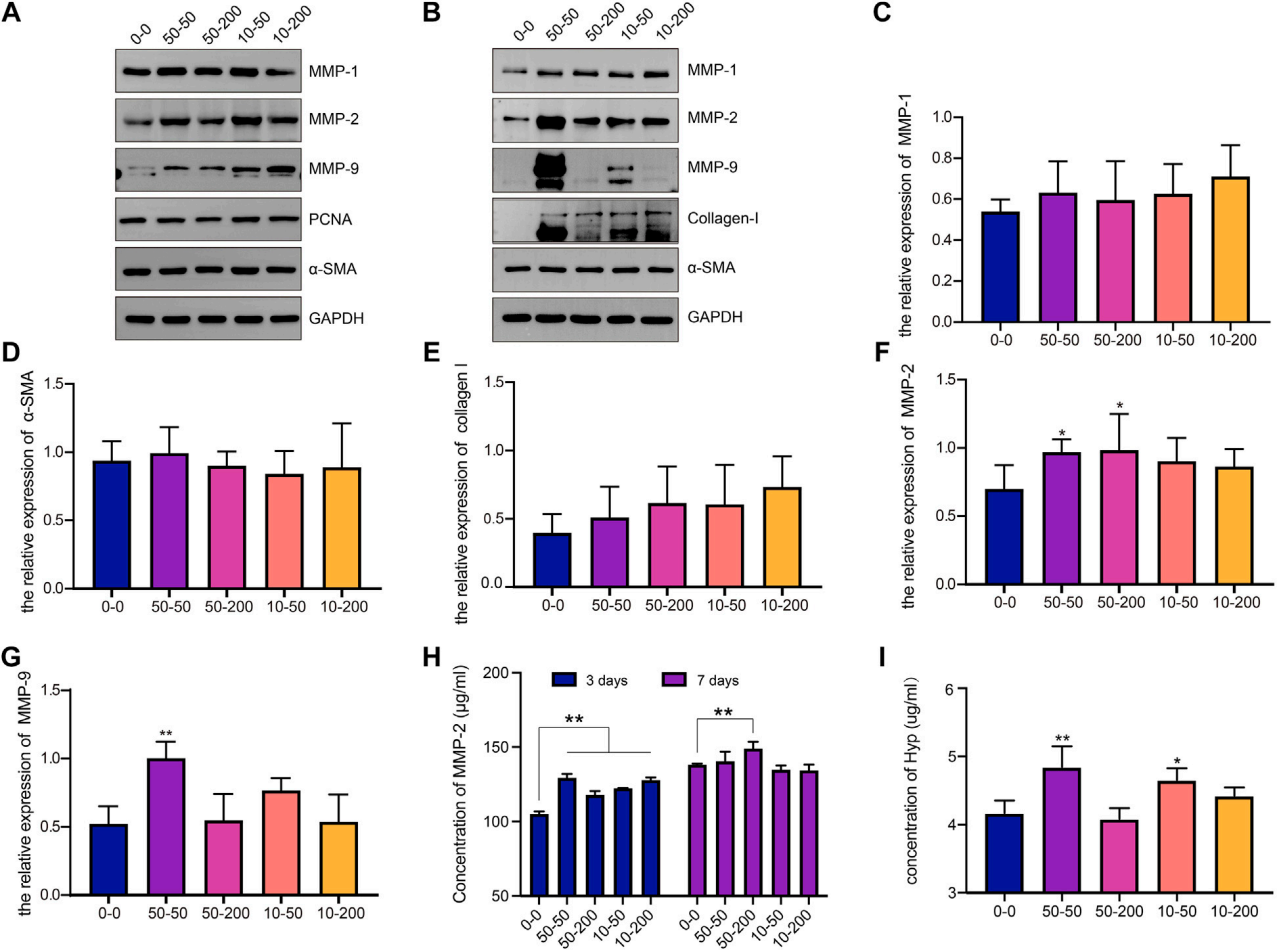
Effects of surface topology on matrix metalloproteinase and collagen secretion of fibroblast. **(A)** Representative WB analysis of MMP-1, MMP-2, MMP-9, PCNA, and α-SMA protein extracts from fibroblasts cultured for 3 days on different samples. One representative blot of three is presented. GAPDH was used as the loading control. **(B)** Representative WB analysis of MMP-1, MMP-2, MMP-9, collagen-I, and α-SMA protein extracts from fibroblasts cultured for 7 days on different samples. One representative blot of three is presented. GAPDH was used as the loading control. **(C)** The relative expression level of MMP-1 in each group. **(D)** The relative expression level of α-SMA in each group. **(E)** The relative expression level of collagen I in each group. **(F)** The relative expression level of MMP-2 in each group. **(G)** The relative expression level of MMP-9 in each group. **(H)** The content of MMP-2 in the supernatant of fibroblasts on the surface of the material was detected by ELISA at 3 and 7 days. **(I)** Hyp content in cell supernatant at 7 days. Data comparisons were implemented utilizing one-way ANOVA, and the values are expressed as mean ± standard deviation (SD). *n* = 5, *p < 0.05, **p < 0.01 *vs* 0–0.

Fibroblasts were cultured on the surface of materials for 3 and 7 days, the total protein was extracted, and the expression of MMP-1, MMP-2, MMP-9, PCNA, α-SMA, and collagen I were detected by western blotting. After the cells were cultured on each material for 3 days, the expression levels of MMP-1 and MMP-2 in groups 50–50 and 10–50 were significantly higher than those in group 0–0, and the expression of MMP-9 in groups 50–200 and 10–200 increased (Figure 5A). After 7 days of cell culture, the expression of MMP-1, α-SMA, and collagen I had no significant difference between the four groove groups and group 0–0 (Figures 5B–E). The expression of MMP-2 and MMP-9 increased in all the experimental groups, but the difference was significant in group 50–50 (Figures 5B,F,G). To further verify the expression of MMPs, the expression of MMP-2 in the cell supernatant was detected by ELISA. As shown in Figure 5H, after 3 days of cell culture, the expression of MMP-2 in the supernatant of cells in the groove group was significantly upregulated, with a statistically significant difference. After 7 days of culture, the amount of MMP-2 in the supernatant of the 50–50 and 10–50 groups increased compared with that of group 0–0; the increase in group 10–50 was statistically significant, and the results were consistent with western blot (WB).

To further explore the ability of cell collagen secretion on the surface of each material, the supernatants of the cells cultured on the surface of each group for 7 days were collected and measured to determine the Hyp content. As shown in Figure 5I, the Hyp content in groups 50–50 and 10–50 was significantly higher than that in group 0–0, and there was no significant difference between groups 50–200 and 10–200. The results show that a microgroove with a small width may promote collagen secretion by cells.

### Effect of Surface Topology on Cell Transcriptome

**FIGURE 6:**
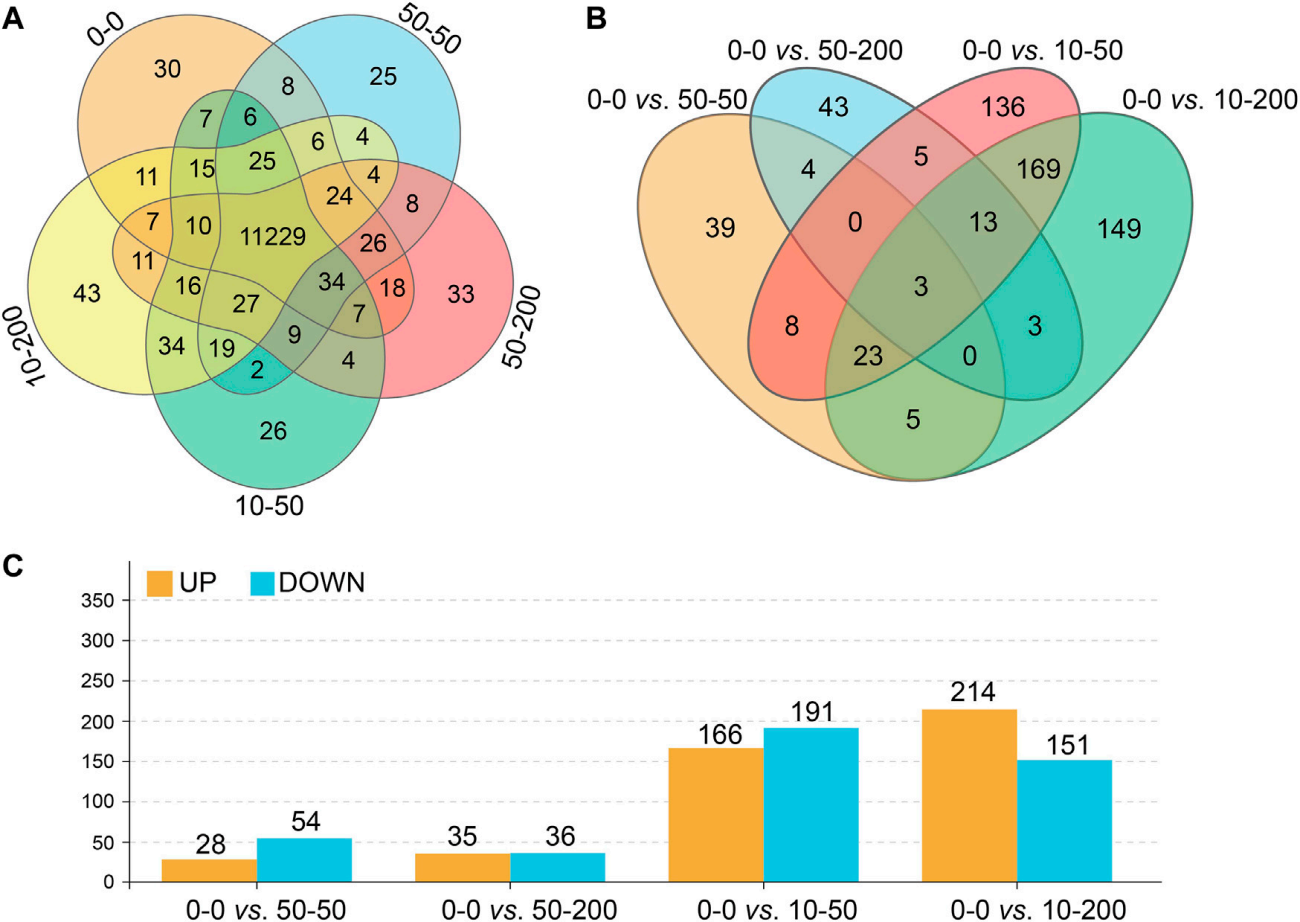
Effect of surface topology on cell transcriptome. **(A)** Total number of gene expressions. **(B)** Differential gene number analysis. **(C)** Up- and downregulation numbers of differential genes.

To further explore the effect of material surface structure on fibroblasts, the changes and differences in genes were studied using RNA-Seq technology, and the sequencing results have been uploaded to the NCBI database (BioProject ID: PRJNA776126). As shown in Figure 6A, the number of genes detected on the surface of each group of materials is shown, 11,229 of which were expressed in five groups. Figures 6B,C show the analysis of genes with significant differences between each group and group 0–0. There were 82 differentially expressed genes in groups 0–0 and 50–50, 28 of which were upregulated and 54 downregulated. There were 71 differentially expressed genes in group 50–200, 35 of which were upregulated and 36 downregulated. There were 357 differentially expressed genes in group 10–50, 166 genes of which were upregulated and 191 downregulated, with 365 differentially expressed genes compared with group 10–200, 214 of which were upregulated and 151 downregulated. The shallower the groove, the more differentially expressed genes between the control group and the experimental group.

**FIGURE 7:**
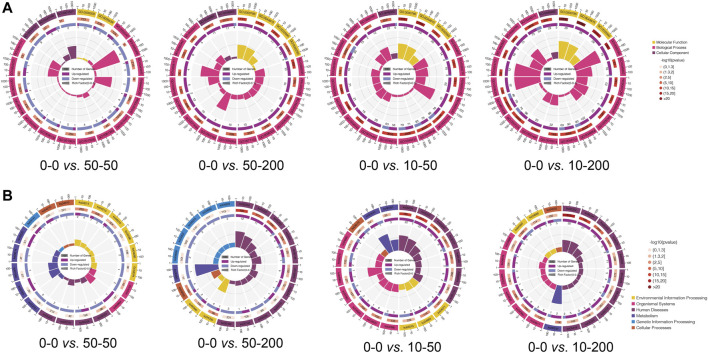
GO and KEGG pathway analysis. **(A)** Circle diagram of top 20 of GO enrichment. **(B)** Circle diagram of top 20 of KEGG enrichment.

We further analyzed the signaling pathways involved in differentially expressed genes by GO and Kyoto Encyclopedia of Genes and Genomes (KEGG) enrichment analyses. As shown in Figure 7, the functions of the top 20 differentially expressed genes and the pathways involved were obtained through an analysis. Figure 7A shows that at least 70% of the differential genes between each group and 0–0 were involved in the cellular biological process. Among the significantly differentially expressed genes in groups 0–0 and 50–50, most of the genes involved in the cell biological processes were related to cell migration, and their expression was mainly downregulated. In addition, the expression of extracellular matrix genes involved in cell composition (GO: 0031012) and microfibril formation (GO: 0001527) were downregulated, and two genes with decreased expressions were related to the regulation of Rho-dependent protein serine/threonine kinase activity. Among the differentially expressed genes in groups 50–200, 10–50, 10–200, and 0–0, the expression of genes related to nuclear composition was significantly upregulated, and the expression of genes related to the androgen signaling pathway was upregulated (GO: 0060765). At 50–200 *vs* 0–0, we observed the upregulation of four genes involved in IL-7–mediated signaling pathway (GO: 0038111), while at 10–50 *vs* 0–0 and 10–200 *vs* 0–0, we found that the expression of megakaryocyte differentiation regulating genes was upregulated (GO: 0045652, GO: 0030219), and the expression of related genes negatively regulated by epigenetic genes was upregulated (GO: 0045814). The expression of amyloid fibril formation–related genes was upregulated (GO: 1990000).

Subsequently, we analyzed the expression of related pathways involved in differential genes through the KEGG pathway. Figure 7B shows that the signals involved in the differential genes between other groups and group 0–0, except group 50–50, were mainly involved in human diseases, while the signal pathways involved in the differential genes between groups 0–0 and 50–50 were environmental information processes and were mainly involved in Ras, Rap1, MAPK, HIF-1, and Fox0 signaling pathways. The signaling pathways involved in cellular processes in differential genes mainly affect focal adhesion (KO04510) and downregulation of actin cytoskeleton (KO04810), which is consistent with our cytoskeleton-staining results. 50–200 vs. 0–0 differential genes were mainly involved in TGF-β (KO04350) and Hippo (KO04390) signaling pathways, and the expression of the signal pathways related to T-cell differentiation (KO04660), Th17 differentiation (KO04659), and Th1/Th2 differentiation (KO04658) was downregulated. The differential genes of the 10–50 vs. 0–0 group affected the signal pathways of TNF (KO04668) and toll-like receptor (KO04620), as well as the expression of focal adhesion (KO04510). The differential genes of 10–200 *vs* 0–0 are mainly involved in the regulation of the TNF signaling pathway (KO04668) and Hippo signaling pathway (KO04390).

**FIGURE 8:**
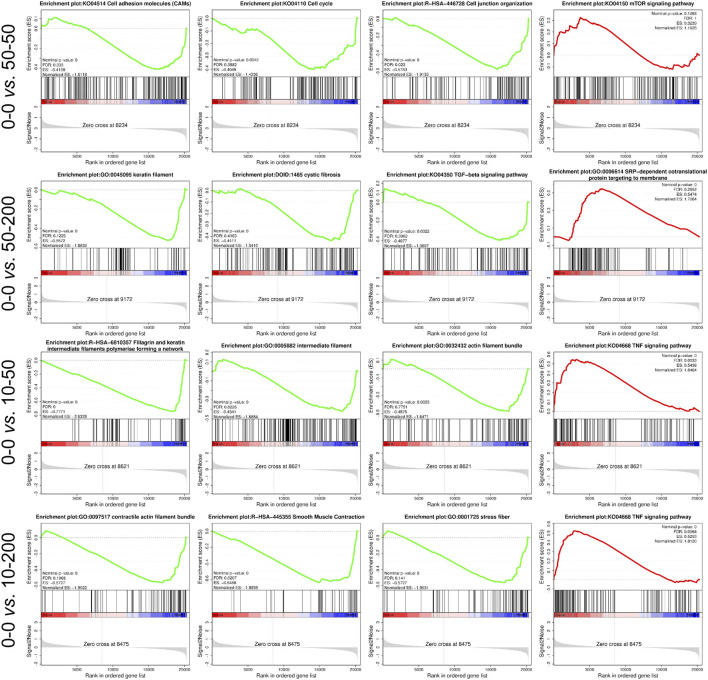
Representation of the GSEA plot.

Furthermore, we performed the GSEA analysis of the transcriptome data. From the representative results, compared with group 0–0, the differential genes in group 50–50 mainly affected the upregulation of cell adhesion molecules, cell cycle, and cell junction organization, while the expression of the mTOR signaling pathway was downregulated. The expression of the 50–200 gene in the keratin membrane, cystic fibrosis, and TGF-β was upregulated compared with that in the control group, whereas the expression of SRP-dependent co-translational protein targeting membrane was downregulated. The gene expression of the signal pathway involved in filaggrin and keratin intermediate filler polymer forming a network and actin filler bundle in group 10–50 was upregulated compared to that in group 0–0, which was consistent with the results of cytoskeleton staining, while the expression of the TNF signaling pathway was downregulated. In group 10–200, the gene expression of the signal pathway involved in contractual actin fiber bundle, smooth muscle contraction, and stress fiber was upregulated, while the expression of the TNF signaling pathway was downregulated (Figure 8).

### Surface Topology of the Material Affects the Deposition of Collagen and the Formation of Capsule

**FIGURE 9:**
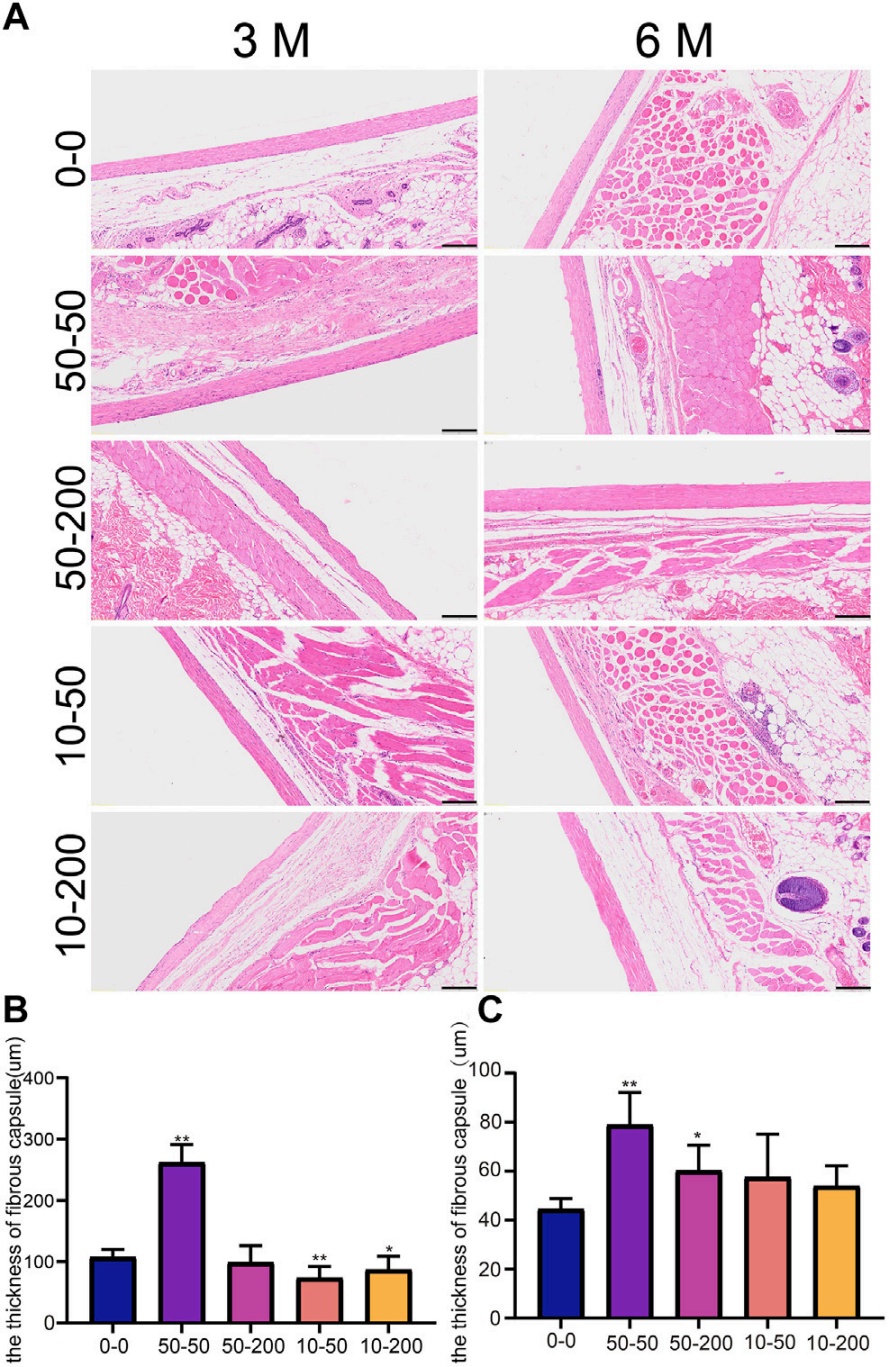
Effect of surface topology on capsule tissue. **(A)** HE staining results of capsule tissue after 3 and 6 months of subcutaneous -implantation of materials in rats. **(B)** Statistical results of capsule thickness at 3 month. **(C)** Statistical results of capsule thickness at 6 month. Data comparisons were implemented utilizing one-way ANOVA, and the values are expressed as mean ± standard deviation (SD). *n* = 10, *p < 0.05, **p < 0.01 vs 0-0.

At 3 months, the capsule around the implanted material of SD rats in group 50–50 was thicker, and additional inflammatory cells could be observed on the contact surface with the material, while there was no significant difference in the capsule thickness between group 50–200 and the smooth group. The capsule thickness of groups 10–50 and 10–200 was slightly lower than that of the 0–0 group (Figure 9B). In addition, we observed a clear dentate structure in the capsules of SD rats implanted with the groove material. Upon measurement, the width of the dentate structure was consistent with the size of the groove on the material surface, indicating that the fine structure on the material surface can cause a change in the tissue morphology. After 6 months of implantation, the capsule thickness of groups 50–50 and 50–200 increased continually, which was significantly different from that of group 0–0. By contrast, there was no significant difference between groups 10–50 and 10–200 and group 0–0, and the tooth-like structure on the tissue almost disappeared at 6 months (Figure 9C).

**FIGURE 10:**
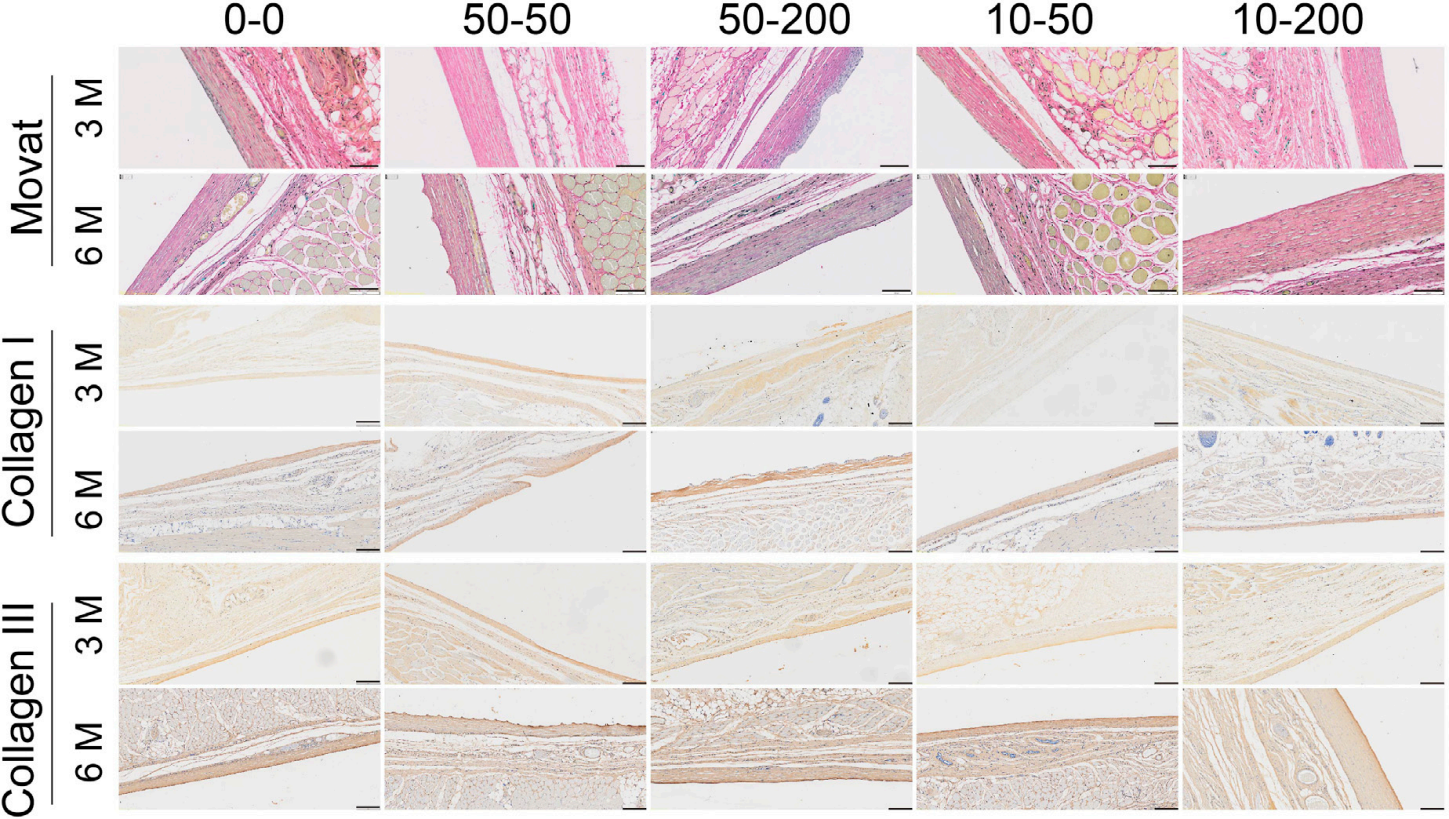
Effect of surface topology on collagen formation and deposition. Representative results of Movat staining, type I collagen, and type III collagen staining (scale bar = 200 μm).

Movat staining can determine a variety of connective tissue components in a tissue section, so it can be used for the pathological study of collagen diseases, atherosclerosis, and mesodermal tumors. Movat staining can dye the nucleus and elastic fibers in black, proteoglycan in blue, collagen fibers and reticular fibers in yellow, and blood cells in bright red (Tabas and Bornfeldt, 2016; Korzinskas et al., 2018). Three months after implantation *in vivo*, yellow-stained collagen fibers and blue-stained proteoglycans were evident around the envelope of materials in groups 0–0, 50–50, and 10–50. A layer of gray elastic fibers was observed around the envelope of materials in groups 50–200 and 10–200, less yellow–stained collagen in the tissue, and a clear dentate structure was visible. At 6 months, the yellow and blue collagen and proteoglycan tissues in each group were significantly increased (Figure 10).

To further explore the tissue composition of the capsule around the material, type I collagen and type III collagen staining were performed. The results of type I collagen staining showed that at 3 months, the expression of type I collagen in each group was lower, but the expression of type I collagen in group 50–50 was significantly higher than that in group 0–0. At 6 months, the expression of type I collagen increased in each group. The results of type III collagen staining showed that there was no significant difference among the groups at 3 months. At 6 months, the expression of type III collagen in group 0–0 was stronger and that in group 10–200 was significantly lower (Figure 10).

**FIGURE 11:**
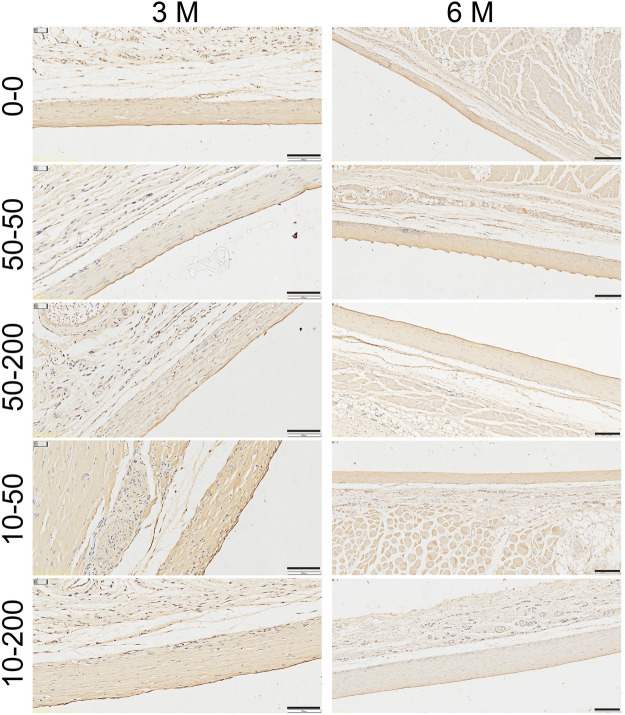
Effect of material surface topology on capsule contracture. Representative results of elastin staining (scale bar = 200 μm).

Elastin is the main component of the elastic fibers. It is an insoluble protein that plays an important role in fibrosis (Kanta, 2016). Immunohistochemical staining showed that the expression of elastin in groups 0–0, 10–50, and 10–200 was strong at 3 months after implantation, while the expression in groups 50–50 and 50–200 was weak. After 6 months, the expression of elastin in group 50–50 was strong (Figure 11). Certain studies have shown that the expression of collagen increases as fibrosis progresses, while the expression of elastin increases significantly at the end of fibrosis, suggesting that reversing fibrosis is more difficult (Chen et al., 2019).

## Discussion

To study the effects of material surface morphology on biocompatibility, microgrooves were constructed on the surface of the silicone implants to change the surface topology, and their biological effects on cells and tissues and their effects on fibrosis were studied. In this study, we successfully constructed four groups of structures with microgrooves of different sizes on the surfaces of the silicone implants. We found that the modification of the surface microgroove structure can significantly increase surface roughness and water contact angle, and there are also significant differences in the type adsorption of proteins by each group. *In vitro* experiments show that at the micron level, the narrower and denser grooves affect the adhesion and proliferation of cells in the early stage and promote the synthesis of collagen in fibroblasts. *In vivo* experiments showed that the tiny structures of surface microgrooves could be observed histologically, and the narrower and denser the grooves (0–0), the more apparent the inflammatory reaction around the grooves that promoted the formation of fibrosis. However, our study only used PDMS as the research material, which varies significantly to clinically applied silicone implants and cannot completely recapitulate the role of prosthesis in the human body.

After the implant is embedded into the body, the protein in the blood begins to adsorb to the material surface within a few seconds, and at this time, the cells cannot adhere to the material surface immediately (Wilson et al., 2005). The adsorption of proteins on the material surface is the initial event of biomaterial body surface contact (Tang et al., 1999; Swartzlander et al., 2015), which determines subsequent processes such as cell growth (Yang et al., 2014), differentiation (Hoshiba et al., 2016), extracellular matrix formation (Abdallah et al., 2017), and body inflammatory response (Othman et al., 2018; Visalakshan et al., 2019). Many factors affect protein adsorption, which are not only related to the properties of materials but also to the surface chemical and physical properties of biomaterials, such as pH, temperature, ionic strength, and concentration of different proteins in the buffer solution (Rabe et al., 2011; Kastantin et al., 2014; Othman et al., 2018). Prior research has revealed that the surface chemistry of biomaterials determines the difference in surface proteomics and further affects the interaction with epithelial cells (Abdallah et al., 2017). Hoshiba et al. (2016) found that poly(2-methoxyethyl acrylate) analogs can control the shape of the cells by changing the adsorption of proteins and promoting the lipogenesis of 3T3-L1 cells. Scopelliti et al. (2010) showed that morphology changes at the surface nano-level had a significant impact on the protein adsorption capacity; in particular, the saturated adsorption capacity increased nonlinearly as nano roughness increased. An increase in the material surface area is conventionally thought to lead to an increase in protein adsorption, but our study results show that the formation of grooves on the surface does not significantly increase the adsorption capacity of tissue proteins but can significantly change the types of adsorbed proteins. The microgrooves on the surface hinder the elution of proteins. Our study found that the change in protein adsorption capacity caused by material surface morphology was mainly concentrated in the early 6 h of adsorption. As adsorption time increased, the adsorption difference caused by surface morphology gradually decreased, and adsorption decreased at 12 h, which may be attributed to the desorption process when protein adsorption reaches saturation. However, our study was limited to a single protein solution rather than a mixed protein solution, and the dynamic adsorption changes of different protein molecules on the material surface could not be observed. Therefore, protein mass spectrometry was carried out to detect the difference in the adsorption of proteins in the extracellular fluid on the surface of microgrooved materials. In contrast to other studies, our results show that the smooth group has the most adsorbed protein types, and the two groups with structures have less adsorbed types, which may be attributed to the difference in hydrophilicity and hydrophobicity on the material surface. Hydrophilicity and hydrophobicity affect the degree of competitive substitution among a variety of proteins (Vogler, 2012), resulting in different types of finally adsorbed proteins, which further causes the reaction of cells and even the body.

Following the development of medical technology, the fibrosis of biomaterial implants, especially capsular contracture after breast augmentation, has drawn the attention of researchers (Barr et al., 2017; Poh et al., 2018; Park et al., 2019; Lombardo et al., 2020). In clinical applications, the use and long-term safety of rough and smooth prostheses have always been controversial among plastic surgeons. At present, rough-surface breast prosthesis implants are largely believed to reduce the incidence of capsule contracture (Liu et al., 2015; Shin et al., 2018). However, our results show that in terms of inducing capsule formation and capsule contracture, materials with rough surfaces are not necessarily better than materials with absolutely smooth surfaces at the micron scale. For example, in this study, the capsule of groups 50–50 and 10–200 were thicker than those of the smooth group, which may be related to the specific size range of the surface texture. At present, there is no agreed standard for the specific classification of prosthesis surface texture. Certain scholars divide the 13 types of prosthesis implants on the market into four types of texture according to roughness and re-entrant features, namely, macro-textured surfaces (Sa > 75 μm), micro-textured surfaces (10 μm < Sa < 75 μm), meso-textured surfaces (Sa < 10 μm), and nano-textured surfaces, namely, smooth surfaces (Sa < 5 μm) (Barr et al., 2017). The International Organization for Standardization (ISO) 14607:2018 classifies breast prosthesis surgical implants into three categories according to the average surface roughness: smooth: <10 μm; microtexture: 10–50 μm; and macrotexture: >50 µm (Wixtrom et al., 2019). Based on the above classification, group 0–0 is considered to be a smooth implant, groups 50–50, 50–200, and 10–50 are micro-textured surfaces, and group 10–200 is meso-textured. Our results show that appropriate micro-textured surfaces may be more effective in reducing capsule formation.

In addition, we found that the narrower the groove surface, the less the stress fibers, which may be because the groove limits the horizontal spread of cells, thus reducing the formation of stress fibers, and the formation of contractile stress fibers marks the formation of myofibroblasts (Hinz et al., 2007). However, there was no significant difference in the expression of α-SMA, mainly because the conditions for the formation of α-SMA–positive myofibroblasts included high TGF-β accumulation, the presence of special ECM proteins, such as ED-A splice variants of fibronectin, and high extracellular stress caused by ECM mechanical properties and cell remodeling activities (Tomasek et al., 2002; Hinz et al., 2007). Therefore, there was no significant difference in the transformation of fibroblasts into myofibroblasts between our experimental group and the control group; however, we observed that the narrower and deeper the groove, the more collagen deposition formed around the material, the thicker the capsule, and the higher the expression of MMP-2 and MMP-9 and the higher the content of Hyp on the surface of the narrower and deeper grooves. The transcriptome results also revealed that the expression of the mTOR signaling pathway was upregulated. Studies have demonstrated the role of the mTOR signaling pathway in tissue fibrosis (Jiao et al., 2017; Saito et al., 2020). Group 50–200 showed strong cell proliferation and low Hyp content. The results of the transcriptome KEGG analysis showed that the expression of the T-cell receptor and Th1/Th2/Th7 differentiation–related signal pathway changed, and the GO results further verified that the expression of IL-17–mediated signal pathway was also different. Certain research results have shown that T cells and helper T cells play a role in fibrotic diseases (Wolfram et al., 2012; Zhang and Zhang, 2020; Adusei et al., 2021), and Th1 cells can produce IFN-γ. This can inhibit fibroblast-induced collagen synthesis to a great extent. In addition, IFN-γ can upregulate the expression of matrix metalloproteinases and degrade extracellular matrix components (Roderfeld et al., 2012). The groove structure on the surface of the material may further affect the process of fibrosis around the material by changing the local immune response. The two groups of grooves with shallow depth (groups 10–50 and 10–200) may regulate the process of fibrosis through the TNF signaling pathway, indicating that the depth and shallowness of materials have different effects on cell biological behavior in different ways. The effect of groove depth on the specific mechanism of tissue fibrosis and the appropriate size that reduces tissue fibrosis around the material require further and more in-depth research.

## Conclusion

We successfully prepared four types of strip microgrooves with different depths and widths on the surface of silicone implants using the photolithography mask method. The formation of microgrooves increased the surface roughness of each group of materials. The narrower and deeper grooves exhibited relatively more roughness and less optimized wettability. Concurrently, compatibility between the cells and tissues was attenuated and was more likely to cause the secretion of collagen in fibroblasts and form a relatively thick envelope around the material.

## Data Availability

The datasets presented in this study can be found in online repositories. The names of the repository/repositories and accession number(s) can be found below: https://www.ncbi.nlm.nih.gov/sra/PRJNA776126.
